# The Effect of the Removal of User Fees for Delivery at Public Health Facilities on Institutional Delivery in Urban Kenya

**DOI:** 10.1007/s10995-017-2408-7

**Published:** 2017-12-29

**Authors:** Lisa M. Calhoun, Ilene S. Speizer, David Guilkey, Elizabeth Bukusi

**Affiliations:** 1 Carolina Population Center, The University of North Carolina at Chapel Hill, Chapel Hill, NC USA; 2 Department of Maternal and Child Health, Gillings School of Global Public Health, The University of North Carolina at Chapel Hill, Chapel Hill, NC USA; 3 Department of Economics, The University of North Carolina at Chapel Hill, Chapel Hill, NC USA; 4 Centre for Microbiology Research, Kenya Medical Research Institute, Nairobi, Kenya

**Keywords:** Institutional delivery, Delivery policy, Urban, Kenya

## Abstract

*Objectives* In 2013, Kenya removed delivery fees at public health facilities in an effort to promote equity in access to health services and address high maternal mortality. This study determines the effect of the policy to remove user fees on institutional delivery in a population-based sample of women from urban Kenya. *Methods* Longitudinal data were collected from a representative sample of 8500 women from five cities in Kenya in 2010 with a follow-up interview in 2014 (response rate 58.9%). Respondents were asked about their most recent birth since 2008 at baseline and 2012 at endline, including the delivery location. Multinomial logistic regression is used, controlling for the temporal time trend and background characteristics, to determine if births which occurred after the national policy change were more likely to occur at a public facility than at home or a private facility. *Results* Multivariate findings show that women were significantly more likely to deliver at a public facility as compared to a private facility after the policy. Among the poor, the results show that poor women were significantly more likely to deliver in a public facility compared to home or a private facility after policy change. *Conclusions for Practice* These findings show Kenya’s progress towards achieving universal access to delivery services and meeting its national development targets. The removal of delivery fees in the public sector is leading to increased use of facilities for delivery among the urban poor; this is an important first step in reducing maternal death.

## Significance

This is the first study from Kenya to use population-based data to demonstrate evidence of the effect of a 2013 policy change removing fees for delivery at public health facilities on delivery practices in urban Kenya.

## Introduction

Globally, an important strategy to reduce maternal and newborn mortality is the promotion of skilled delivery, often attained through institutional delivery (Moyer et al. 2013). In 2013, in an effort to address continued high maternal mortality in Kenya and promote equity in access to health services, President Uhuru Kenyatta announced the abolishment of delivery fees in public health facilities. This announcement expanded a 2007 national policy which removed delivery fees at primary-level public facilities. In addition, following the 2013 general election, nearly concurrent to the removal of delivery fees, Kenya established a decentralized government under which health service provision and promotion was transferred to 47 county governments. Under the decentralized approach, the central government maintained control over national referral hospitals, health policy decisions and the development of regulations and standards for service delivery (Williamson and Mulaki 2015; Nyikuri et al. 2015; The Constitution of Kenya [Kenya] 2010). The decision to remove delivery fees at public facilities was timely given that Kenya was not on track for meeting its Millennium Development Goals 4 (infant and child health) and 5 (maternal health); this policy change contributes to addressing these deficits.

Skilled attendance at delivery and promoting facility delivery is a means to reduce maternal death by having skilled attendants trained to handle uncomplicated deliveries and who can identify complications for further referral. At the national level, there have been recent improvements in skilled attendance at delivery in Kenya with an increase from 44% in 2008/2009 to 62% in 2014 (Kenya National Bureau of Statistics (KNBS) and ICF Macro 2015). Similarly, institutional delivery increased from 40% in 2003 to 61% in 2014 (Kenya National Bureau of Statistics (KNBS) and ICF Macro 2015). Despite this increasing trend, disparities exist in utilization of facilities. Facility delivery is more common among young, urban, educated and wealthy women in Kenya (Kenya National Bureau of Statistics (KNBS) and ICF Macro 2015; Kitui et al. 2013; Fotso et al. 2009a).

With rapid urbanization occurring globally and in Kenya, there is an increased focus on understanding health outcomes in cities and within specific populations in cities, such as the urban poor. Urban areas are typically characterized by a higher quality and more diverse health infrastructure, including a robust private sector, as compared to rural areas (Vlahov et al. 2007; Montgomery 2009). However, despite this urban health sector advantage, it is not equitably used. The urban poor often lack access to health services due to cost, transportation and time (Vlahov et al. 2007; Montgomery 2009; Fotso et al. 2008; APHRC (African Population and Health Research Center) 2002). Further, the facilities which the urban poor have access to may be of lower quality in that they lack appropriate staffing, equipment, and medications for delivery services (Fotso et al. 2008, 2009b). Finally, in some cases, the urban poor have worse health outcomes than their rural counterparts (Vlahov et al. 2007; Montgomery 2009; Fotso et al. 2008; APHRC (African Population and Health Research Center) 2002).

Previous studies have shown increases in facility-based deliveries after the removal of delivery fees (Dzakpasu et al. 2012; Penfold et al. 2007; Ridde et al. 2011; Steinhardt et al. 2011; Witter et al. 2010; McKinnon et al. 2015), though proponents of user fees cite that these fees are important for revenue generation in addition to improving quality of care (Collins et al. 1996). A recent systematic review highlighted methodological limitations with much of the evidence on this topic, including failure to account for sources of bias, including secular time trends, the lack of information on key contextual issues, and challenges arising from use of facility-based data such as the representativeness of the sample and the quality of the routine health data used in the analyses (Dzakpasu et al. 2014). One of the stronger articles in the review comes from a multi-country study that used a difference-in-differences approach to investigate the effect of a policy change on institutional delivery; the study included the 2007 policy change in Kenya removing fees for delivery at primary-level public health facilities (McKinnon et al. 2015). Though the paper finds a significant effect of policy changes on institutional delivery across all countries, it is a multi-country analysis and does not provide results specific to Kenya (McKinnon et al. 2015). Furthermore, there is a lack of evidence on the success of implementation and operationalization of the 2007 policy as well as indications that the policy may not have been adhered to (Chuma et al. 2009; Chuma and Maina 2013; Ministry of Public Health and Sanitation 2008).

The effect of the 2013 removal of delivery fees at all public health facilities in Kenya, not just at primary-level public facilities, has not been rigorously studied. The Health Policy Project (HPP), which is a partnership between the USAID and the Government of Kenya, assessed the impact and implementation of the policy through a survey of public and faith-based health facilities in 15 counties. HPP found that there was a 26% increase in the number of deliveries occurring in primary-level public facilities after 1 year whereas there was only a 2% increase at faith-based dispensaries and health centers in the same time period (Maina and Kirigia 2015). A population-based survey is needed to determine if this national policy change has resulted in an increase in use of public sector facilities for delivery and a reduction in home deliveries.

This study fills this gap using a population-based sample of women with births pre- and post-policy introduction from five cities in Kenya. This provides a natural experiment to examine the impact of the Government of Kenya policy to remove user fees on delivery behaviors. A secondary objective is to investigate if the urban poor were more likely to deliver at public health facilities after the policy change.

## Methods

In 2009, the Bill & Melinda Gates Foundation funded the Urban Reproductive Health Initiative (URHI) with the goal of increasing modern contraceptive use in urban areas in four countries: Kenya, Nigeria, Senegal and India. As part of the URHI, the Measurement, Learning & Evaluation (MLE) project, led by the Carolina Population Center at the University of North Carolina at Chapel Hill, was funded to undertake rigorous impact evaluation of the four country programs. The evaluation design included data collection from a longitudinal sample of women and health facilities in specific urban sites in each country.

In Kenya, baseline data were collected in 2010 in Kakamega, Kisumu, Machakos, Mombasa, and Nairobi from households and women. A two-stage sampling design was used to select a representative sample of women from each city; at the first level, the 2009 Population and Housing Census frame was used to identify and randomly select primary sampling units (PSU). A household listing was then completed for all selected PSUs and, at the second stage, a random sample of 30 households was selected in each selected PSU. In selected households, all women ages 15–49 were eligible for interview. The total baseline sample size across the five cities is 8932 women.

Women who were usual residents at baseline were eligible for participation in the endline survey (n = 8850). In 2014, eligible longitudinal respondents were tracked and those who were found in a study city were approached for interview. Upon giving consent for participation at each survey wave, longitudinal respondents were asked about sociodemographic characteristics, fertility, use and access to maternal and child health services, family planning use, media exposure and migration. Of the women eligible at endline, 5217 were successfully interviewed yielding an endline response rate of 58.9%. The study design and surveys have been described previously (Measurement, Learning & Evaluation (MLE) Project, Tupange and KNBS 2011; Measurement, Learning & Evaluation (MLE) Project 2015). All study procedures were approved by the Institutional Review Board at the University of North Carolina at Chapel Hill and the Kenya Medical Research Institute Ethical Review Committee.

The analysis sample for this paper is restricted to the 2793 women in the baseline survey who had a birth since 2008 and the 1332 women in the endline survey who had a birth since 2012; this yields a total sample of 4125 unweighted births for this analysis (3922 births weighted). Among the analysis sample, 579 women (unweighted) had a recent birth at both baseline and endline (yielding 1158 births). Also of interest, 284 women (unweighted) had a birth both before and after the delivery policy change. A small number of women with missing data on place of delivery (endline n = 13, baseline n = 27), education (endline n = 5), marital status (baseline n = 8) and religion (baseline n = 1) were omitted from this analysis.

The dependent variable for this analysis is place of delivery. Women who had given birth since 2008 for baseline or since 2012 for endline were asked where they gave birth to their most recent child. Responses were categorized as public facility, private facility or home/other location. Public facilities include government hospitals, health centers, and dispensaries. Private facilities include faith-based hospitals and clinics, private hospitals and clinics, and nursing or maternity homes. Home includes women who delivered at their own home, someone else’s home or on the way to a health facility. Other responses include traditional birth attendant, traditional healer, and community midwife.

The key independent variable is the timing of the birth which indicates if the birth occurred prior to or after the launch of the policy on June 1, 2013. Births which occurred between June 2013 and January 2015 were coded 1 and all births prior to June 2013 were coded zero. We also control for the linear time trend in timing of births in months before or after the introduction of the policy with births that occurred in June 2013 coded as zero.

Models control for the following sociodemographic characteristics: education (no education, incomplete primary, complete primary, secondary or higher); marital status (ever married/in union, never married/in union); age (15–24, 25–34, 35+ years); city (Nairobi, Mombasa, Kisumu, Machakos, Kakamega); religion (Protestant/Christian, Muslim, no religion, Catholic); and birth order (first birth, second birth, third or higher order birth). Births that were reported at baseline were assigned the baseline sociodemographic characteristics, and births that were reported at endline were assigned the endline characteristics.

In addition, a three category wealth variable is included: poorest/poor, middle, rich/richest. The dummies are calculated based on wealth indices calculated at baseline and at endline using a list of 21 items including household assets and materials; the methodology used to create the indices was based on the methods devised by Filmer and Pritchett (2001). The household sample was divided into quintiles ranked from the poorest to the richest. Women were assigned a wealth score based on the household in which they resided. In this analysis, women in the poorest and poor wealth categories were combined to represent the poor and those in the rich and richest categories were combined to represent the rich.

### Statistical Analysis

Univariate and bivariate analyses are weighted using the baseline or endline weights based on which survey round the birth is from. Descriptive analysis of births to women who have multiple births in the analysis sample utilize the baseline weights. Multivariate analyses are unweighted and adjust for the clustered sample design. All analyses were performed using Stata version 14.

This natural experiment allows for the investigation into the casual effect of the change in policy on place of delivery among the full analysis sample of births and also among the poor, controlling for sociodemographic characteristics and the number of months before or after the policy. Determination of exposure to the policy was exogenous to the individual. We perform multinomial logistic regression to estimate the effect of the policy change on institutional delivery where the model takes the following form:

$$\ln \left[ {\frac{{P\left( {{D_{ti}}=k} \right)}}{{P\left( {{D_{ti}}=l} \right)}}} \right]={X_{ti}}{\beta _k}+Dur\_{t_i}{\delta _k}+~{P_{ti}}{\gamma _k}+{\varepsilon _{ti}}~$$

We are fitting the log-odds that woman i (i = 1, 2, …, N) from time t delivered at location *k* relative to location *l* as a linear function of covariates. *D* is a categorical outcome variable of whether the delivery occurred in a public health facility, private health facility or at home/other. The background characteristics, such as age, education and religion, are represented by *X. Dur_t* represents the linear number of months before and after the policy change. The *P* represents whether the birth occurred before or after the policy change removing fees for delivery at public facilities. The final term in the equation is an individual level error term.

The results are discussed as relative risk ratios, or the anti-log of the parameter estimates. Thus results presented can be interpreted as the likelihood of the outcome relative to the reference category. For instance, the column labeled ‘public versus private’ in Table 4 can be interpreted as the likelihood of delivery at a public facility relative to a private facility.

## Results

Table 1 presents the characteristics of women who had a live birth since 2008 for the baseline survey and 2012 for the endline survey. As expected, the endline sample is older, has had more children, and a higher percentage have been ever married. About 42 and 47% of women were in the poorest/poor wealth category at baseline and endline, respectively. The sample was predominantly Christian, with about two-thirds being Protestant/Christian and one-quarter being Catholic; Muslims accounted for 10% of both samples. Among the baseline sample of births, about 16% delivered their most recent birth at home and 46 and 38% delivered at public and private facilities, respectively. In the endline sample, only 9% delivered at home, 41% delivered at a public facility and 49% delivered at a private facility. Among women who delivered at a facility, at both baseline and endline, approximately 65% of women delivered at a public or private hospital (not shown). All births in the baseline sample occurred before the 2013 policy change whereas about 54% of the endline births occurred after the initiation of the policy.

**Table 1:** Characteristics of women who had a live birth since 2008 for the baseline survey and since 2012 for the endline survey

Characteristic	Baseline (2010) distribution	Endline (2014) distribution
Education		
No education	3.62	4.26
Incomplete primary	15.68	11.97
Complete primary	29.80	23.35
Secondary or higher	50.90	60.42
Marital status		
Ever married/in union	90.71	93.32
Age group (years)		
15–24	45.11	17.22
25–34	46.26	68.95
35+	8.63	13.83
City		
Nairobi	70.81	69.35
Mombasa	19.26	22.35
Kisumu	6.55	4.96
Machakos	1.19	1.29
Kakamega	2.19	2.05
Wealth group		
Poorest/poor	42.37	47.20
Middle	25.59	16.55
Rich/richest	32.05	36.25
Religion		
Protestant/Christian	62.90	66.80
Muslim	10.24	9.51
No religion	2.22	1.26
Catholic	24.65	22.44
Birth order		
First birth	43.54	27.75
Second birth	27.08	34.66
Third or higher order birth	29.38	37.59
Place of delivery		
Public	46.12	41.32
Private	38.18	49.37
Home/other	15.70	9.31
Birth occurred after change in delivery policy		
Yes	0.00	53.69
No	100.00	46.31
Weighted N	2592	1570
Unweighted N	2793	1332

Home includes women who delivered at their own home, someone else’s home or on the way to the hospital. Other responses include traditional birth attendant, traditional healer, and community midwife

Table 2 presents the cross tabulation of place of delivery by the timing of birth (pre-policy or post-policy) by survey wave. In the endline sample, weighted percentages show that about equal percentages of women delivered their most recent birth at a public facility before and after the policy, at 41 and 42%, respectively. There was a large increase in private sector delivery after the policy introduction, from 45 to 53%. Home deliveries decreased by about 9% points from 14% before the policy to 5% after the policy introduction. Table 2 also shows the place of delivery by timing of the birth and survey wave among the poor. In the endline sample of the poor, public sector delivery increases from 40 to 46% after the introduction of the policy and private sector deliveries increased from 36 to 45%. There was a corresponding decrease in home deliveries from 24 to 10% after the policy change.

**Table 2:** Place of delivery by exposure to delivery policy among women who had a live birth since 2008 for the baseline survey and since 2012 for the endline survey

Facility type	All births						All births among the poor^c^					
	Baseline sample^a^			Endline sample^b^			Baseline sample^a^			Endline sample^b^		
	Before introduction of policy	After introduction of policy	Total	Before introduction of policy	After introduction of policy	Total	Before introduction of policy	After introduction of policy	Total	Before introduction of policy	After introduction of policy	Total
Public	46.12	0.00	46.12	40.58	41.96	41.32	44.63	0.00	44.63	39.97	45.62	43.21
Private	38.18	0.00	38.18	45.29	52.89	49.37	29.80	0.00	29.80	35.77	44.83	40.96
Home/other	15.70	0.00	15.70	14.13	5.15	9.31	25.56	0.00	25.56	24.25	9.55	15.83
Total	100.00	0.00	100.00	100.00	100.00	100.00	100.00	0.00	100.00	100.00	100.00	100.00
Weighted N	2592	0	2592	727	843	1570	1098	0	1098	317	424	741
Unweighted N	2793	0	2793	664	668	1332	1474	0	1474	365	348	713

^a^Baseline weights were used for descriptive analyses of the baseline sample

^b^Endline weights were used for descriptive analyses of the endline sample

^c^The poor is the lowest two wealth quintiles

Table 3 shows further analysis of the 284 women who had a birth both before and after the 2013 policy introduction. Among women who delivered their pre-policy birth at home/other, 42% delivered their post-policy birth at a public facility and 28% at a private facility. Among women who delivered their pre-policy birth at a private facility, about 44% delivered their post-policy birth at a public facility, 55% remained at a private facility and only 1% delivered at home. The majority of women who delivered their pre-policy birth at a public facility delivered their post-policy birth at a public facility as well (55%), about 32% switched to a private facility and 12% delivered their post-policy birth at home. The Pearson Chi square for this cross tabulation is 39.06 and has a p value of 0.02. Overall, a smaller percentage of post-policy births were delivered at home than pre-policy.

**Table 3:** Place of delivery for pre-policy and post-policy births among women who had multiple births (unweighted N = 284)

Before introduction of policy	After introduction of policy					
	Public	Private	Home/other	Total (%)	Weighted N	Unweighted N
Public	55.40	32.18	12.42	100.00	108	138
Private	44.15	54.83	1.02	100.00	124	74
Home/other	42.08	28.39	29.52	100.00	39	72
Total (%)	48.34	41.96	9.70	100.00		
Weighted N	131	114	26		272	
Unweighted N	159	82	43			284

Pearson Chi square = 39.06, p = 0.02

Table 4 shows the multivariate multinomial logistic regression results of the effect of the policy and covariates on place of delivery where three comparisons are presented: public facility to home delivery, private facility to home delivery, and public facility to private facility delivery. The results for the linear trend indicate a significantly positive effect for the comparison of public versus home and private versus home while the public versus private comparison indicates a slight movement towards private delivery but this effect is only significant at the 10% level and not the 5% level. Births that occurred after the policy change were not significantly more likely to occur in a public or private facility as compared to home. Women who were less educated, poorer and younger were less likely to deliver in any facility as compared to home, as shown in the comparisons for public versus home and private versus home. Lower parity women were more likely to deliver in a public or private facility as compared to home. Distinctions were seen by city which may be reflective of the diversity of the health sector and differences in cultural norms in each city. Compared to Kakamega, facility delivery is more common in Nairobi and Kisumu whereas it is less common in Mombasa.

The policy change dummy did not have a significant effect in the comparison of public or private facility to home. However, the results for the public versus private comparison show that women who delivered after the policy change were 1.3 times more likely to deliver in a public facility than a private facility compared to women who delivered before the policy. Women who were younger, poorer and have incomplete or complete primary education as compared to those with secondary and above were more likely to deliver in a public facility than a private facility. Women who have ever been married or in union, or live in Nairobi, Mombasa, Kisumu, or Machakos as compared to Kakamega were less likely to deliver in a public facility than a private facility.

**Table 4:** Relative risk ratios from multinomial logistic regression models of the estimated effect of delivery policy launch on place of delivery among women who had a birth since 2008 for the baseline survey and since 2012 for the endline survey (unweighted N = 4125)

Variables	Public versus home			Private versus home			Public versus private		
	RRR	SE	p Value	RRR	SE	p Value	RRR	SE	p Value
Birth occurred after policy change	1.281	0.257	0.216	0.962	0.214	0.862	1.332	0.192	0.046
Education									
No education	0.240	0.066	0.000	0.391	0.143	0.010	0.613	0.195	0.123
Incomplete primary	0.284	0.038	0.000	0.211	0.035	0.000	1.348	0.174	0.020
Complete primary	0.484	0.062	0.000	0.367	0.054	0.000	1.317	0.129	0.005

Secondary or higher (ref)									
Marital status									
Ever married/in union	1.257	0.211	0.171	1.825	0.389	0.005	0.689	0.102	0.012
Age group (years)									
15–24	0.647	0.120	0.019	0.360	0.074	0.000	1.800	0.290	0.000
25–34	0.940	0.151	0.699	0.662	0.109	0.012	1.419	0.185	0.007
35+ (ref)									
City									
Nairobi	1.407	0.249	0.054	6.917	1.346	0.000	0.203	0.030	0.000
Mombasa	0.654	0.122	0.022	2.077	0.439	0.001	0.315	0.053	0.000
Kisumu	1.100	0.204	0.606	3.297	0.719	0.000	0.334	0.053	0.000
Machakos	0.822	0.167	0.335	1.140	0.260	0.564	0.721	0.118	0.045
Kakamega (ref)									
Wealth group									
Poorest/poor	0.272	0.046	0.000	0.101	0.018	0.000	2.680	0.277	0.000
Middle	0.598	0.119	0.010	0.301	0.062	0.000	1.987	0.243	0.000
Rich/richest (ref)									
Religion									
Protestant/Christian	1.042	0.116	0.710	1.050	0.159	0.745	0.992	0.112	0.946
Muslim	1.321	0.277	0.184	1.348	0.364	0.268	0.980	0.209	0.924
No religion	1.279	0.478	0.511	1.151	0.528	0.760	1.111	0.429	0.784
Catholic (ref)									
Birth order									
First birth	3.315	0.508	0.000	3.494	0.587	0.000	0.949	0.110	0.650
Second birth	1.989	0.255	0.000	1.985	0.287	0.000	1.002	0.109	0.986

Third or higher order birth (ref)									
Linear time trend	1.020	0.003	0.000	1.025	0.003	0.000	0.995	0.002	0.069

Given that a goal of the policy was to ensure equitable access to health facilities, particularly among poor and vulnerable populations, we estimated the same multinomial logistic regression specification for a sample restricted to the poor (see Table 5 where poor refers to the two lowest wealth quintiles). The results show that poor women who delivered after the policy change were 1.6 times more likely to deliver in a public facility than at home and 1.8 times more likely to deliver in a public facility than a private facility compared to women who delivered before the policy change. All other variables show similar results to the full sample model.

**Table 5:** Relative risk ratios from multinomial logistic regression models of the estimated effect of delivery policy launch on place of delivery among women in the poorest and poor wealth quintiles who had a birth since 2008 for the baseline survey and since 2012 for the endline survey (unweighted n = 2187)

	Public versus home			Private versus home			Public versus private		
Variables	RRR	SE	p Value	RRR	SE	p Value	RRR	SE	p Value
Birth occurred after policy change	1.577	0.343	0.036	0.885	0.243	0.657	1.782	0.402	0.010
Education									
No education	0.310	0.087	0.000	0.515	0.214	0.110	0.602	0.218	0.162
Incomplete primary	0.318	0.046	0.000	0.349	0.066	0.000	0.912	0.148	0.569
Complete primary	0.541	0.076	0.000	0.526	0.093	0.000	1.028	0.150	0.847

Secondary or higher (ref)									
Marital status									
Ever married/in union	0.951	0.188	0.798	1.102	0.291	0.713	0.863	0.196	0.516
Age group (years)									
15–24	0.813	0.175	0.337	0.603	0.177	0.084	1.349	0.383	0.292
25–34	1.077	0.201	0.690	0.897	0.216	0.653	1.201	0.286	0.442
35 + (ref)									
City									
Nairobi	1.374	0.276	0.114	17.547	5.040	0.000	0.078	0.021	0.000
Mombasa	0.661	0.133	0.039	3.850	1.252	0.000	0.172	0.052	0.000
Kisumu	1.109	0.242	0.635	8.550	2.586	0.000	0.130	0.036	0.000
Machakos	0.835	0.178	0.396	1.307	0.509	0.491	0.638	0.240	0.233
Kakamega (ref)									
Religion									
Protestant/Christian	1.095	0.147	0.499	0.911	0.186	0.649	1.202	0.217	0.309
Muslim	1.284	0.297	0.280	1.502	0.523	0.243	0.854	0.259	0.604
No religion	1.796	0.659	0.110	0.408	0.284	0.198	4.404	2.839	0.021
Catholic (ref)									
Birth order									
First birth	3.217	0.607	0.000	2.923	0.637	0.000	1.100	0.212	0.620
Second birth	2.082	0.314	0.000	1.950	0.365	0.000	1.068	0.186	0.707

Third or higher order birth (ref)									
Linear time trend	1.015	0.003	0.000	1.026	0.004	0.000	0.989	0.003	0.002

As a robustness check, we ran models without parity as an explanatory variable since parity is potentially endogenous to place of delivery choice and including endogenous regressors without a correction for their endogeneity could bias not only the parity effect but the effects of other variables that are correlated with parity. Results for the policy variable were very similar with and without parity and so we kept parity in the final model specification.

## Discussion

In this natural experiment where exposure to a national policy removing fees for delivery services at public health facilities is randomly determined, we provide the causal effect of the policy on institutional delivery. Our study found that women from five cities in Kenya were significantly more likely to deliver at a public facility as compared to a private facility after the policy change; however, we did not find a significant effect of the policy on delivery at either a public or private facility versus at home in the full sample. Among a sample of multiparous women whom had a birth both before and after the policy change, we see that a significant percentage of women switched from delivering at home to a public facility for their post-policy birth; this is suggestive of policy effects as prior studies illustrate that higher-order births are more likely to be delivered at home (Fotso et al. 2008, 2009b) and post-policy births are by nature higher-order. Our results among the poor show that poor women were significantly more likely to deliver in a public facility compared to home or a private facility after the policy change. Given that a key goal of the policy is to increase equitable access to services, our findings among the urban poor show that the policy has reached some of the women most in need.

With increasing attention at the global level to encourage skilled attendance at delivery, policies that promote institutional delivery, such as the Kenya policy, are important steps to improve maternal and newborn survival. Our findings show that about 86% of women in this study in five urban sites in Kenya delivered their most recent birth in a health facility before the policy and about 95% after the policy; these findings are slightly higher than the 2014 KDHS which found that about 82% of urban women delivered their most recent birth in a health facility (Kenya National Bureau of Statistics (KNBS) and ICF Macro 2015). An objective of the policy is to address disparities in access to institutional delivery, particularly for the poor who are often disproportionately affected by the cost of institutional delivery (Kenya National Bureau of Statistics (KNBS) and ICF Macro 2015; Hotchkiss et al. 2005; Stekelenburg et al. 2004; Witter et al. 2007). Our findings show nearly a 15% point increase in institutional delivery among the urban poor in our endline sample after the introduction of the policy. Controlling for time trends, the multivariate results show that the change in delivery policy has increased use of public facilities for delivery among the poor, thus highlighting the policy’s progress towards equitable access.

Existing evidence on the success of the 2013 policy is limited and inconsistent. A paper by Maina and Kirigia (2015) shows increases in public sector deliveries post-policy, yet this finding is based on facility surveys from a subset of counties. Conversely, Tomedi et al. (2015) collected data from 29 rural primary-level health facilities in Machakos County and did not show a significant increase in skilled birth attendant deliveries in a 7 month period after introduction of the policy. These two studies had a limited geographic range and highlight the need for population-based evidence. Finally, qualitative findings from interviews with providers and facility administrators in Malindi in early 2014 highlight challenges in policy implementation which affected the quality of care provided including stock-outs, staff shortages, and delays in reimbursement of funds from the government (Lang’at and Mwanri 2015). Our study builds on this existing facility-level research and provides more substantial evidence of the success of the policy.

This paper has a few limitations worth mentioning. First, the data for this analysis come from a longitudinal study which means that the sample of women ages over time and may have more births with every survey wave; these factors may be associated with home delivery (Fotso et al. 2008, 2009b). Further, given the longitudinal nature of the study, there was attrition between survey waves, and the study lost a disproportionate number of younger, unmarried women. Additionally, the survey tool only captures information about the most recent birth, meaning that we do not have information on place of delivery for previous births. This may be important as delivery location for previous births may be associated with location for subsequent births.

In addition, a number of national policy changes were implemented in a short time frame. The policy change to remove delivery fees and that to decentralize the government were put into effect within a few months of each other, both of which may have implications on quality of care. Provision of health services, including staffing and management of health facilities, was decentralized to county governments and many implementation challenges were experienced during this transition (Williamson and Mulaki 2015; Nyikuri et al. 2015). As shown in the study by Lang’at and Mwanri (2015), the removal of fees for delivery resulted in a reduction in the quality of care available at facilities. This study did not collect information on the quality of care from the health facilities where the respondent delivered, and therefore we do not know if there were changes in quality of care after the policy change which may affect utilization of facilities for delivery (Dzakpasu et al. 2014). Finally, in 2007 there was a national policy announced to remove fees for delivery at primary-level public health facilities. Little information is available on the implementation, scale and impact of this 2007 policy and therefore it is not possible to disentangle how it affects the 2013 policy change and our results. Notably, a key difference between the 2007 and 2013 policy change is that the 2007 removal of fees was only for delivery at primary-level facilities, yet our urban data show that when women deliver in a facility, they tend to choose to deliver at a higher level facility. This is counter to evidence from rural Tanzania (Kruk et al. 2009).

Future studies and program strategies should focus on understanding and reducing barriers to facility delivery among the urban poor. Urban women, faced with a large, diversified health sector, may make choices about facility delivery based on a number of factors. Future research can link women to the facilities they actually go to for delivery and investigate factors associated with this facility choice by looking at the distance, quality and costs of delivery at selected facilities. In particular, quality of care merits further investigation due to concerns that the increase in demand for services could result in poorer quality of care over time (Dzakpasu et al. 2014; Lang’at and Mwanri 2015). In addition, qualitative studies can be carried out to understand unique barriers to delivery for poor women in urban settings above and beyond those barriers captured in standardized quantitative questionnaires. Finally, a large dataset with both urban and rural domains capturing pre- and post-policy births should be analyzed to determine the effectiveness of the policy in these different environments.

In conclusion, these findings show Kenya’s progress towards achieving universal access to delivery services and meeting its national development targets. The removal of user fees for delivery in the public sector is leading to increased use of facilities for delivery among the urban poor; this is an important first step in reducing MMR for this population.
